# Women’s Health: Most Common Physiologic and Pathologic Cutaneous Manifestations During Pregnancy

**DOI:** 10.7759/cureus.16539

**Published:** 2021-07-21

**Authors:** Azhar M Barnawi, Ghassan M Barnawi, Awadh M Alamri

**Affiliations:** 1 Family Medicine, Armed Forces Hospital, Prince Mansour Military Hospital for Community Medicine, Taif, SAU; 2 Dermatology, College of Medicine at King Saud bin Abdulaziz University for Health Sciences, King Abdulaziz Medical City, Jeddah, SAU

**Keywords:** cutaneous, changes, common, pregnancy, a systematic review

## Abstract

Pregnant women are susceptible to various physiological and pathological skin and body habitus changes during pregnancy due to the alterations that occur in a multi-organ-system fashion. Pregnancy can be the onset of different dermatological diseases and can exacerbate pre-existing cutaneous conditions. Moreover, management of dermatologic diseases during pregnancy might be challenging as it requires special attention to both mother and fetus. We aim to assess the most common cutaneous changes and conditions that occur during pregnancy by reviewing the previous studies conducted on this subject.

The medical literature was explored through PubMed and Google scholar databases starting from 2015 to 2021. The included searching terms were a combination of "Cutaneous changes and pregnancy," Dermal conditions and pregnancy," Pregnancy-associated dermal conditions," and "Dermatological changes and pregnant women." The inclusion criteria included original articles conducted on pregnant women and full text- articles.

A total of 134 articles were obtained, 11 articles were eligible for the inclusion criteria. The 11 studies included a total number of 14,813 pregnant women and covered four countries. The most common cutaneous conditions experienced by pregnant women were primarily physiological skin changes, pregnancy-specific dermatopathologies, and exacerbations of other common skin diseases.

All in all, this systematic review concluded that pregnant women are more vulnerable to various dermatological conditions during pregnancy. These changes were more commonly physiological such as hyperpigmentations. However, pathological pregnancy-specific skin conditions and exacerbations of pre-existing dermatoses like atopic eruptions were also reported.

## Introduction and background

Pregnancy is associated with multiple physiological changes involving different organ systems like the endocrine, vascular, metabolic, and immune systems of gravid women resulting in several cutaneous changes which can be physiological or pathological [1]. These conditions are due to physiological changes, specific dermatoses of pregnancy, and other common pregnancy non-specific skin diseases in pregnancy [2].

Physiological skin changes during pregnancy primarily involve alterations in the degree of skin pigmentation and skin laxity mainly due to the effect of the elevated hormones [3]. Pregnancy-specific dermatologic pathologies involve impetigo herpetiformis, cholestasis of pregnancy, prurigo of pregnancy, pruritic folliculitis, pruritic urticarial papules, and plaques of pregnancy. Moreover, other existed skin conditions, such as psoriasis and atopic dermatitis, may worsen and present with flares during pregnancy [4,5]. However, improvement in some dermatological skin diseases during pregnancy can also be seen [6].

Pregnant women are mostly aware of most of the common physiological skin changes that occur during pregnancy, and they commonly tend to seek medical advice for actual new-onset pathological skin conditions or exacerbations of pre-existing inflammatory skin disorders [2]. However, the presentation of many dermatological diseases during pregnancy can vary and may have atypical presentation; for example, itching can occur in one among five normal pregnancies, but it can be the presenting symptom of several pregnancy dermatoses. Therefore, the accurate diagnosis of various dermal conditions during pregnancy can be difficult [2]. In addition, pregnancy can change the management of these common skin conditions and make it more challenging; these challenges are mostly related to the safety and harm of medications and other interventions like laser therapy to the developing fetus [2]. Therefore, we conducted this review of the literature to evaluate and discuss the various pregnancy-related dermatological conditions.

## Review

Method

The PRISMA checklist guidance for systematic review and meta-analysis [7] was followed to write this systematic review. The two databases: PubMed and Google scholar databases were revised searching for literature articles. The eligible research articles between 2015 and 2021 were selected.

Search strategy

Several keywords were used for searching purposes, including a combination of “Cutaneous changes and pregnancy,” “Dermal conditions and pregnancy,” “pregnancy-associated dermal conditions,” and “Dermatological changes and pregnant women.” All the titles and abstracts produced from this primary exploration were revised thoroughly to prevent missing potential studies. The findings were then examined to choose only original research articles evaluating the dermatological changes during pregnancy. All full-text articles written in English were defined as articles of relevance, which were then included in the second stage.

Eligibility criteria

**Figure 1:**
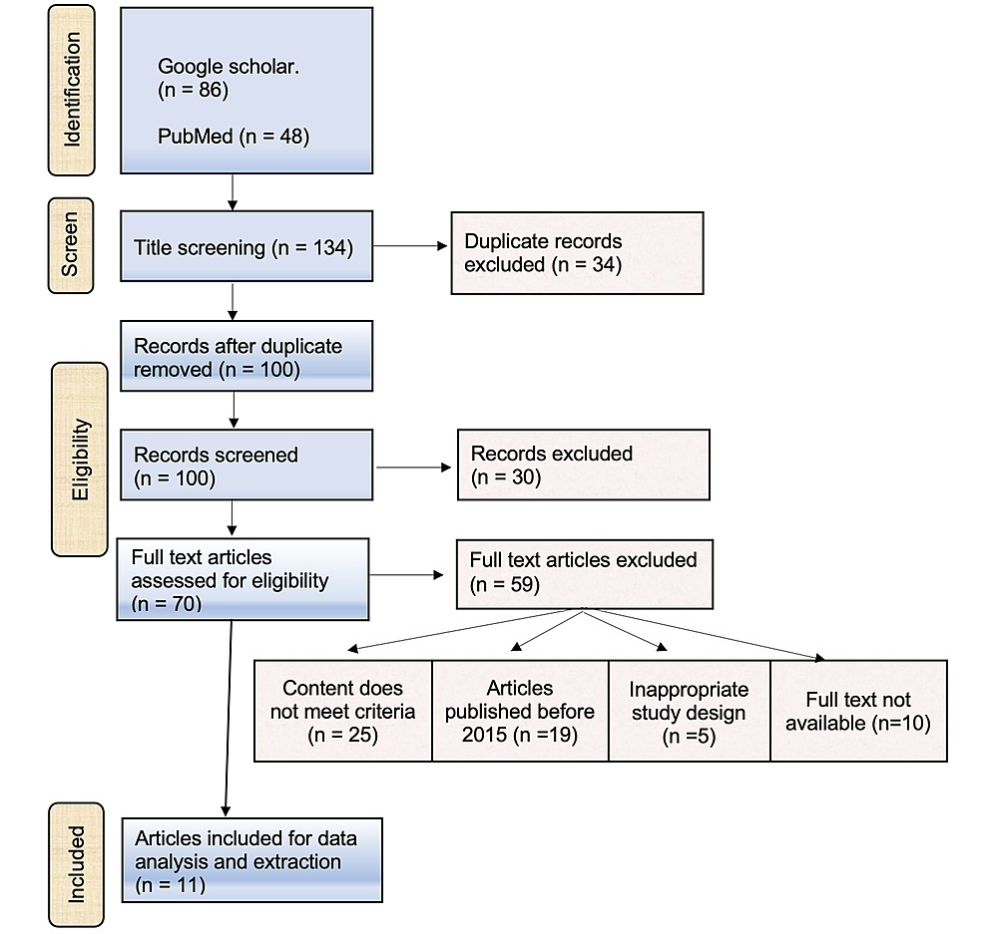
Planning of eligible criteria

The second step was deciding on the inclusion criteria to select the eligible studies. Abstracts were assessed manually to select the relevant studies for revision. The inclusion criteria were studies conducted on pregnant women and investigated the physiological changes and cutaneous conditions during pregnancy. Reviews and studies that had incomplete or overlapped data were excluded. Also, unavailable full-text articles or inappropriate study designs were excluded. The full description of the search strategy is shown in Figure 1.

Data review and analysis

A specially designed excel sheet was used for data extraction. The chosen data from eligible research articles were then revised via the excel sheet. Any research articles published by one research group examining similar variables were reviewed for any potential duplication.

Results

This systematic review included 11 articles that met the eligibility criteria and were published between 2015 and 2021 (Table 1) [8-18]. In regard to the study designs of included articles, there were two cross-sectional studies [14,18], three cross-sectional were observational studies [10,16,17], one observational [8], one prospective observational cohort [9], one retrospective [11], and one prospective [13] whereas one study did not specify the design [12]. The total number of participants in the 11 studies was 14,813 participants, the least number of subjects included in a study was 100 pregnant women [12], and the largest number included was 9,679 [8]. The included studies were conducted in only four countries; one study in Nigeria [9], one study in Libya [13], one study in Brazil [18], whereas the remaining eight studies were all in India [8,10-12,15-17]. The overall major dermatological conditions during pregnancy were physiological skin changes [11-18] and pregnancy-specific dermatopathologies [8,9]. However, only one study reported that STDs and other infectious skin disorders were the major presenting dermatosis during pregnancy [10].

**Table 1:** Summary of included studies

Author and Publication year	Study design	Population, Sample size	Country	The most common manifestation/change	Results and main findings
Deora et al. 2021 [8]	Observational	-9,679 pregnant women	India	- Specific dermatoses 40.85%	*164(1.69%) had various skin disorders *67 (40.85%) of them had specific dermatoses of pregnancy *Prurigo of pregnancy (49.12%) of the cases with specific dermatoses, pruritic urticarial plaques and papules of pregnancy (43.93%), pruritus gravidarum (6.06%), Pemphigoid gestationalis (1.2%)
Ayanlowo et al. 2020 [9]	Prospective observational cohort	-296 pregnant women	Nigeria (West Nigeria)	-Signs of dermatoses 85.5%	*Itching 42.2%, signs of dermatoses 85.5% *3.7% Specific dermatoses; Atopic eruption of pregnancy (AEP) (72.72%%) and pruritic urticarial papules and plaques of pregnancy were seen in (27.27%) *The most common non-specific pregnancy dermatoses were acne vulgaris (43.6%), superficial fungal infections (40.2%) and melasma (8.6%). *A background history of atopic dermatitis was significantly associated with AEP. *skin conditions are common in pregnancy; fatal pregnancy dermatoses were not seen in this study.
Choudhary et al. 2020 [10]	Cross-sectional observational	-1,425 pregnant women	India	-Infection, infestations, and sexually transmitted diseases 53.5%	* Pathological dermatoses (19.29%), Infection, infestations and sexually transmitted diseases (53.5%), pregnancy-specific dermatoses (24.7%), acne and folliculitis (7.6%), non-specific itching (5.8%), other conditions (10.9%) * A knowledge of the profile of dermatoses during pregnancy is essential to plan preventive measures, care of the mother and the child
Agarwal et al. 2020 [11]	Retrospective	-308 pregnant women	India	-Pigmentary changes 97.35%	*98.05% presented with physiological skin changes of pregnancy; pigmentary changes (97.35%) *38.31% had pregnancy-specific dermatosis, 60.06% had pregnancy non-specific dermatosis. *The most common pregnancy-specific dermatosis was atopic eruption (44.8%), polymorphic eruption of pregnancy (32.2%) * In non-specific dermatoses, infectious diseases were more common * Lower socioeconomic strata and overcrowding may be the reasons behind a large number of infectious dermatoses that we saw in our study.
Sharma et al. 2019 [12]	-----	-100 pregnant women	India	-All women experienced physiological skin changes of pregnancy; hyperpigmentation with linea nigra 82%	*100% had physiological skin changes of pregnancy *2% had specific dermatoses of pregnancy; 2% cases had Pruritic Urticarial Papules and Plaques of Pregnancy *14% presented with other dermatoses associated with pregnancy *The most common infectious dermatosis affected by pregnancy in this study group was vulvovaginal candidiasis (5 cases). *82% hyperpigmentation with linea nigra, followed by 68% changes of connective tissue (steriae gravidarum) *Skin changes are common during pregnancy and are usually benign and self-limiting. Pregnancy specific dermatoses though few are symptomatic can be associated with severe fetal outcomes such as fetal distress, stillbirth, and premature birth *Differentiating physiological skin changes of pregnancy from pregnancy-specific dermatosis/ other disease conditions can avoid unnecessary investigations and management and aid in better patient care and counseling
ElFaituri 2019 [13]	Prospective	-200 pregnant women	Libya	-Physiological skin changes; 17%	* 71% coincidental or alteration in pre-existing diseases, 17% physiological changes, 12% specific dermatoses *54% of pregnancy dermatoses occurred during the third trimester *Hyperpigmentation and strieagravidarum represented the main physiological changes (17%). *Specific pregnancy dermatoses were present in 12%, these were intrahepatic cholestasis of pregnancy (4%), an atopic eruption of pregnancy (3%), pemphigoid gestationis (3%) and polymorphic eruption of pregnancy (2%). *Fetal complications including fetal mortality were reported with pemphigoid gestationis (30%) and intrahepatic cholestasis of pregnancy (25%). *Most of the reported pregnancy dermatoses were benign with no adverse effect on the fetus. Pemphigoid gestationis and intrahepatic cholestasis of pregnancy can be a source of significant fetal risk.
Chakraborty et al. 2019 [14]	Cross-sectional	-400 pregnant women with cutaneous manifestations	India	-All patients experienced physiological changes; Linea nigra (86.8%)	*Most common pigmentary changes; Linea nigra (86.8%), areolar hyperpigmentation (68.2%), melasma (29.5%) *Most common connective tissue changes; striae gravidarum (75.2%) *8.75% specific dermatoses; prurigo of pregnancy (8%) followed by pruritic urticarial papules and plaques of pregnancy (0.5%). *36.75% Non-specific dermatoses; The commonest was dermatophyte infection *Prurigo of pregnancy was more frequently recorded in multigravida patients and more frequently in the third trimester of pregnancy * the specific dermatoses of pregnancy, which are not a rare entity, can be a source of significant distress to the pregnant female and need timely therapeutic intervention *Physicians should distinguish between physiological skin changes and specific dermatoses of pregnancy for better patient care
Bangaru et al. 2019 [15]	Prospective cross-sectional	-700 pregnant women	India	-All pregnant experienced physiological skin changes; Linea nigra (87.14%)	*Physiological skin changes; 87.14% Linea nigra *8.28% Specific dermatoses; (62.06%) atopic eczema, (31.03%) pruritic urticarial papules and plaques of pregnancy * Many skin changes that occur during pregnancy are physiological require no treatment. *Knowledge about pregnancy specific dermatoses is necessary as dermatoses specific to pregnancy can affect the pregnancy and the fetus
Meena & Gehlot 2018 [16]	Observational cross-sectional	-200 pregnant women	India	-Physiological changes (98%); hyperpigmentation (94.49%)	* 98% had physiological changes; (94.49%) had hyperpigmentation, (88.5%) had linea nigra, (76.5%) had striae distensae, (53.5%) had secondary areola *5% had specific dermatoses; (5%) had pruritic urticarial papules and plaques of pregnancy, (2%) had pruritic folliculitis, (2%) had eczema *Pregnant women are prone to various cutaneous manifestation during pregnancy. A detailed history and awareness of clinical presentation is helpful for confirmation of diagnosis and most appropriate laboratory evaluation is helpful to diminish the maternal and fetal morbidity
Panicker et al. 2017 [17]	Observational cross-sectional	-600 pregnant women	India	-Physiological changes (99%); hyperpigmentation (87.6%)	*Physiological changes 99%; hyperpigmentation (87.6%), striae gravidarum (72.8%), * 2% Specific dermatoses the most common being pruritic urticarial papules and plaques of pregnancy (1.3%) *Pregnant women are prone to suffer from a wide range of dermatological problems apart from specific dermatoses of pregnancy
Fernandes & Amaral 2015 [18]	Cross-sectional	-905 pregnant women	Brazil	-Physiological skin changes (88.95%); Linea nigra (54.75%)	* Physiological skin changes (88.95%); Linea nigra (54.75%), Melasma (54.03%) stretch marks (46.96%) *8.72% specific dermatoses; atopic eruption (70.88%) * Physiological changes were seen more in the 3rd quarter, as well as the specific dermatoses

Physiological skin changes are the predominantly recognized dermatologic manifestations of pregnancy as eight studies reported [11-18], three of which discovered that all included pregnant women had physiological skin changes [12,14,15]. On the other hand, these physiological changes varied in prevalence in two different studies, which showed a range of 17% [13] to 99% [17]. Pregnancy-related abnormal skin pigmentation is the most recognized physiological dermatologic-related sign during pregnancy (87.6%-98.05%) [16,17], followed by hyperpigmentation with linea nigra (82%) [12], linea nigra (54.75%-87.14%) [15,17], striae gravidarum or stretch marks 46.96% [17] to 2.8% [18], and melasma (54.03%) [18].

Dermatosis of pregnancy or pregnancy-specific dermatosis is a major cause of morbidity during pregnancy and its prevalence has ranged from 2% [12,17] to 40.85% [8], whereas one study [9] reported that these pregnancy-specific dermatoses can affect up to 85.5% of pregnant women. Dermatosis specific to pregnancy included mainly, but not limited to, prurigo of pregnancy (8%-47.12%) [8,14], pruritic urticarial plaques, and papules of pregnancy (0.5%-43.93%) [8,14], and atopic eruption of pregnancy (4%-72.72%) [9,13].

Discussion

During pregnancy, several changes occur to optimize fetal and maternal well-being which should eventually result in the delivery of a healthy baby, these changes include changes in the function of the endocrine system to regulate the various hormone secretions, immune system, and the various metabolic pathways that ensure delivery of nutrients and metabolic fuel to the fetus. Some of these changes contribute to a spectrum of physiological and pathological physical changes seen during pregnancy, such as the development of various skin conditions [4]. In the current systematic review, we found that skin pigment disorders account for the most common physiological skin conditions in gravid women, whereas atopic eruptions were the major dermatoses specific to pregnancy.

Physiological dermatological changes in pregnancy involve pigmentary changes such as hyperpigmentation, which occurs among 90% of pregnant women. It usually occurs in a localized area and may be due to the regional differences in the density of melanocytes within the epidermal layer of the skin. However, generalized hyperpigmentation can occasionally occur [19,20]. A dark line that forms from the mid suprapubic area to the umbilicus called linea nigra is a common finding in pregnant women as well [4]. Melasma, which is known as the mask of pregnancy, is a common, pregnancy-specific pigmentation caused by excess estrogen. It is more obvious in women with darker skin tones and occurs in topographical areas such as cheeks, upper lip, and forehead. It mostly appears in the second trimester of pregnancy [21] and can affect up to 50% to 70% of women [22]; in this systematic review, however, only one study [18] reported that half of the pregnant women experienced melasma. Though some studies have shown that these pigmentary lesions could affect a wide range of pregnant women [13,17], The prevalence of such pigmentary skin lesions during pregnancy can truly vary depending on various factors, but some studies have reported that almost every single gravid woman may complain from pigmentary skin changes during pregnancy [12,14,15].

Besides pigmentary changes, striae gravidarum or stretch marks are common structural skin changes commonly seen in pregnancy due to the rapid change in weight and the effects of elevated hormones on the integrity of collagen and other skin constituents [17,18]. Striae gravidarum are structural skin changes, and they occur in up to 90% of pregnant women in the third trimester [21,23]. They occur commonly at the lower abdomen as the connective tissue beneath the skin tear during pregnancy due to excessive stretch, which helps accommodate the rapidly growing fetus [4].

Dermatoses specific to pregnancy are skin pathologies that commonly occur in pregnancy or exacerbations of pre-existed dermatologic diseases seen in gravid women. The most common pregnancy-specific dermatosis was the atopic eruption of pregnancy which was seen in 4% to 72.72% of women with pregnancy-associated dermatosis [9,13] and it was also reported in other four studies as well but with lower frequency [9,11,13,18]. Prurigo of pregnancy can be considered in the second rank as it has been reported in up to 47% [8,14], and there were two studies that reported the presence of this condition [8,14]. In the third rank, pruritic urticarial plaques and papules of pregnancy have a range between 0.5% and 43.93% [8,14].

Atopic eruption of pregnancy is a benign pruritic condition that is characterized by popular lesions or eczema in patients with a history of atopic dermatitis or predisposition to atopic dermatitis or even with new-onset atopic dermatitis during pregnancy. Its prevalence was stated to be in the range of 5%-20% [5]; however, the included studies reported a much higher prevalence (4%-72.72%). Prurigo of pregnancy belongs to the pregnancy-specific dermal diseases and this condition involves different variants; the mild and most common variant, which is characterized by localized lesions only, and the generalized variant, which is known as popular dermatitis [3]. Pruritic urticarial plaques and papules of pregnancy are other pregnancy-specific dermatological diseases. It is a pruritic inflammatory cutaneous disease with an incidence of one in 160 pregnant women [24]. Although it was reported that Pruritic urticarial plaques and papules of pregnancy are the most common pregnancy-specific dermatoses [24], the current systematic review showed that atopic eruption was the most common dermatoses specific to pregnancy.

This systematic review has few limitations, such as the majority of the studies included were from India; however, these studies were included as they met the inclusion criteria.

## Conclusions

Pregnant women are more prone to several cutaneous conditions during pregnancy, such as physiological skin changes, pregnancy-specific dermatoses, and flares of pre-existing chronic skin conditions. The most common physiological skin changes are hyperpigmentary changes, whereas atopic eruption is the most common dermatoses form. Eczematous eruptions have been the most common exacerbation of a chronic skin condition during pregnancy. These pathological skin conditions can be a source of considerable distress to pregnant women and may warrant immediate interventions, though their diagnosis and management can be challenging and require a thorough knowledge of their different presentations and special treatments to ensure maternal and fetal safety. Therefore, a physician's knowledge about the profile of these various dermatoses during pregnancy is necessary in order to plan preventive measures and provide comprehensive care for the mother and her baby.
